# Environmental regulation of root growth angle in cereal crops

**DOI:** 10.1007/s11104-026-08535-2

**Published:** 2026-04-22

**Authors:** Marco Lombardi, Riccardo Fusi, Atish Bansod, Maxwell Asiedu, Gizem Dimlioglu, Gwendolyn K. Kirschner, Rahul A. Bhosale

**Affiliations:** 1 Department of Agroecology, Climate and Water, Aarhus University, 8830 Tjele, Denmark; 2 School of Biosciences, University of Nottingham, Nottingham, LE12 5RD Nottingham, UK; 3 James Hutton Institute, Invergowrie, DD2 5DA UK; 4 The Waite Research Institute and The School of Agriculture, Food and Wine, The University of Adelaide, Waite Campus, Urrbrae, South Australia, Australia

**Keywords:** Root growth angle, Water and nutrient uptake, Environmental stresses, Hormonal regulation, Crop resilience

## Abstract

**Background:**

In recent years, the root system has emerged as a critical determinant of crop resilience and productivity under soil-related environmental stresses. Among root architectural traits, the root angle is particularly important because it shapes the depth and spatial distribution of roots, thereby influencing access to water and nutrients. A steeper angle can promote deeper soil exploration, whereas a shallower angle can improve topsoil foraging; each can confer advantages under specific stress conditions, including drought, flooding, salinity, and nutrient deficiency.

**Scope:**

This review explores the ecological relevance of the root angle, illustrated by adaptations observed in cereal crops across Australia, a continent characterized by diverse climatic and edaphic conditions. We then review the current understanding of how crop plants adjust root angle in response to water-related stresses (drought, flooding, osmotic and salinity) and nutrient (nitrate and phosphate) deficiency on root system and molecular level. We examine the underlying mechanisms and physiological responses, highlighting the roles of key hormonal pathways, particularly auxin, abscisic acid and ethylene and explore how cell wall mechanics potentially acts as a converging point for developmental and environmental signals to fi ne-tune root angle.

**Conclusions:**

Root angle is emerging as a critical trait for improving crop resilience and productivity under increasingly variable environmental conditions, and its ecological relevance, genetic variability, and responsiveness to environmental cues underscore its potential as a breeding target. Despite these insights, major knowledge gaps remain, particularly in understanding how multiple tropic responses coordinate under combined stresses and how these responses vary across root classes.

## Climate change, water and nutrient availability and the food security challenge

One of the most pressing challenges of the twenty-first century is ensuring food security for a global population projected to grow by two billion over the next 30 years. This challenge is intensified by climate change, which is expected to increase the frequency and severity of extreme environmental events, placing additional stress on crop species that are poorly adapted to such fluctuating conditions (Intergovernmental Panel on Climate Change, 2022). Projections indicate that global yields of major cereals such as wheat, maize, and rice could decline by 10–25% by 2050 due to climate change (Jägermeyr et al. 2021). Key environmental factors include reduced soil fertility, increased acidity, compaction, hypoxia, water stress, and suboptimal soil temperatures, which all are perceived at the root-soil interface (George et al. 2024). Consequently, root traits are increasingly recognised as critical targets for breeding climate-resilient crops (Lombardi et al. 2021; Quint et al. 2024; Kohli et al. 2022; Ndoye et al. 2022).

In this review, we focus on root angle, a key architectural trait with a strong potential for improving stress resilience and productivity. We discuss its genetic variability and give examples for the ecological relevance of the root angle in the context of Australia, which represents a continent with diverse climatic gradients and soil conditions. We then explore how soil-related environmental stresses, including drought, flooding, salt stress and nutrient availability-related stresses, regulate the root angle and review recent findings on the molecular mechanisms underlying these responses. Additionally, we highlight the emerging role of the cell wall as a converging point for developmental (gravitropic and anti-gravitropic) and environmental signals that shape root angle plasticity. Finally, we discuss how multiple simultaneous stresses may regulate root angle and highlight open questions that must be addressed to fully exploit this trait in breeding programs aimed at improving stress resilience and productivity under climate change.

## Root angle: a potential target for improving stress resilience and productivity

Substantial progress has been made in improving aboveground traits; however, the root system remains an underexploited target for enhancing crop performance under stress (Lynch et al. 2022).Roots anchor the plant, absorb water and nutrients, mediate symbiotic interactions, and store carbohydrates. These functions depend on root system architecture (RSA), which refers to the three-dimensional configuration of roots in the soil (Mehra et al. 2025). Among RSA traits, root angle is particularly important because it determines the direction of growth, resulting in either a steeper (more vertical) or shallower (more horizontal) RSA. The root angle is primarily established in relation to gravity and is referred to as the gravitropic set-point angle (Digby and Firn 1995). The gravitropic set-point angle arises from a dynamic equilibrium between gravitropic (downward) and antigravitropic (upward) growth response (Fusi et al. 2022; Kirschner et al. 2024).

**Fig. 1:**
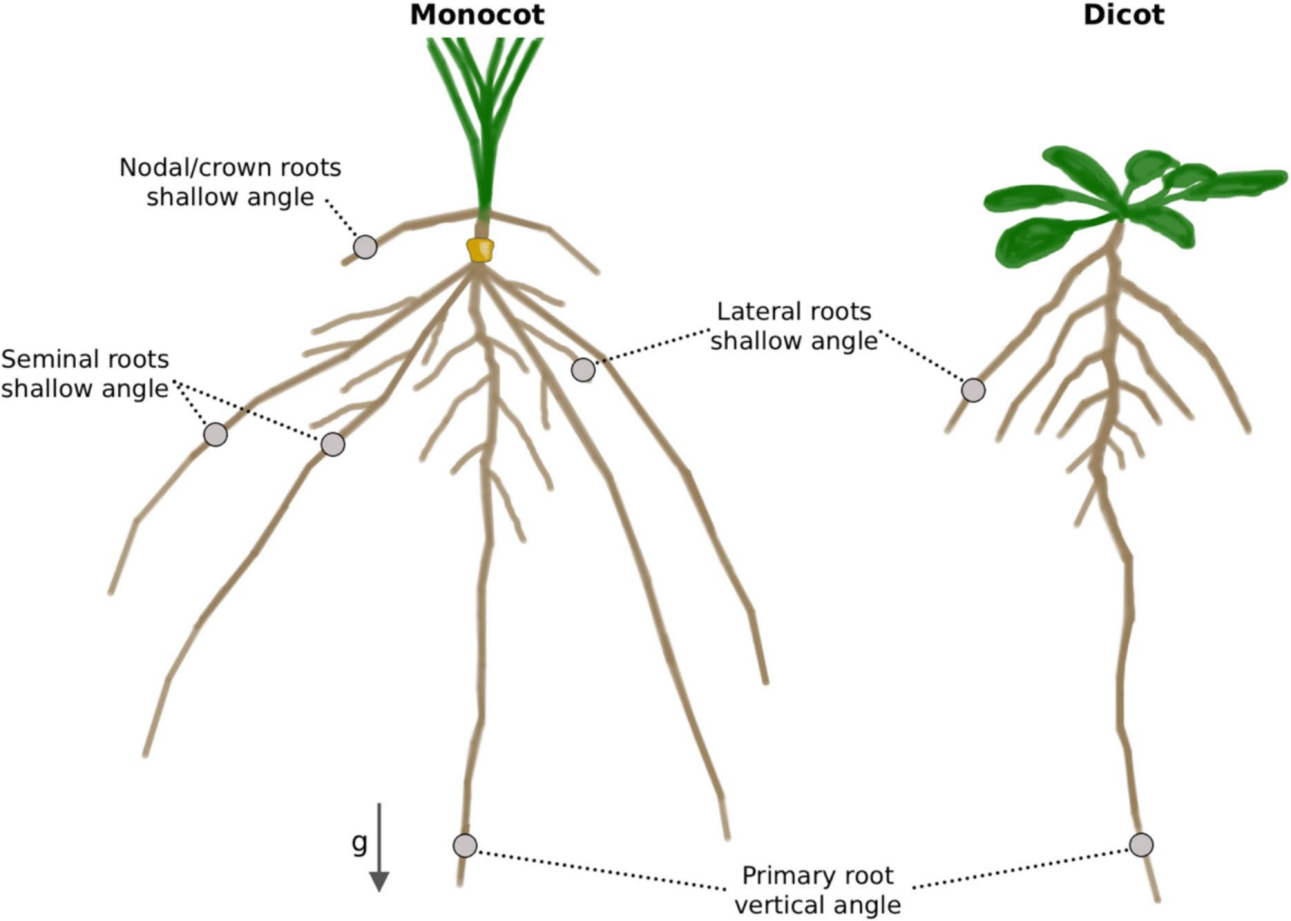
**Different root types in monocots and dicots exhibit distinct root angles.** Primary roots are established in the embryo in both monocots and dicots. They are the first to emerge after germination and grow vertically (towards the vector of gravity, g). Seminal roots are embryogenic roots characteristic for monocots, which grow non-vertically, i.e. in a shallower angle than the primary roots. First and second order lateral roots grow out from all root types and explore the soil space more horizontally. Adventitious roots (nodal or crown roots) arise from the stem in monocots

Different root classes exhibit distinct angles, influencing their ability to explore soil layers and acquire both mobile and immobile resources (Fig. 1). For example, steep root angles enable deeper soil exploration, enhancing access to water and mobile nutrients like nitrate, while shallow root angle promotes topsoil foraging, improving access to immobile nutrients such as phosphate (Lynch 2019). These functional differences make root angle a promising breeding target for optimising resource use and improving resilience to environmental stress, thereby reducing reliance on external fertiliser inputs.

For cereals, which are mostly grown in temperate and semi-arid regions, two root ideotypes have been proposed to address these contrasting soil resource distributions: (**a**) steep, cheap, and deep to improve nitrogen and water uptake from deeper soil layers. This ideotype features a steep root angle, deep root length density, and reduced lateral branching, making it suitable for drought-prone environments (Lynch 2013; Ndoye et al. 2022); and (**b**) topsoil foraging: optimized for phosphorus and potassium uptake from shallow soil layers. This ideotype is characterised by a shallow root angle, dense lateral root branching, and a high proportion of roots near the soil surface (Van Der Bom et al. 2020).

However, these ideotypes may not be universally optimal under all conditions. Soil constraints such as acidity, salinity, compaction, and hypoxia can limit root growth and function (Van Der Bom et al. 2020). Therefore, a more nuanced understanding of root angle plasticity and its regulation is needed to design root systems that are adaptable to diverse and changing environments.

## Root angle shows genetic variability across cereal crops

Root angle is a polygenic trait controlled by multiple unique and conserved genes and genomics regions across major cereals such as wheat, barley and rice (Boudiar et al. 2020; Voss-Fels et al. 2018). This trait shows substantial genetic variation and high heritability, making it an attractive target for breeding programs (Alemu et al. 2021; Boudiar et al. 2020). Below, we focus on wheat as an example for brevity, but similar patterns of genetic variability have been reported in other cereals (Ren et al. 2022; Schneider et al. 2022; Teramoto and Uga 2024; Aldiss et al. 2025).

Most insights into root angle variability in wheat comes from studies on commercial durum wheat cultivars and landraces. For example, Sanguineti et al. (2007) observed moderate variation and high heritability for the “Spread of Root angle (SRA)” trait, which showed weak correlation with other root architecture traits, indicating independent genetic control (Sanguineti et al. 2007). Although no significant differences were observed among germplasm pools from Italy, ICARDA, and North America, Alahmad et al. (2019) found that drought tolerant ICARDA accessions showed significantly steeper root angles compared to elite Australian cultivars (Alahmad et al. 2019). This suggests that selection for aboveground traits and yield may indirectly shape root phenotypes adapted to different water availability gradients.

Landraces, with their broader genetic diversity, represent an untapped resource for novel root angle alleles. For instance, collections from Ethiopia (Alemu et al. 2021) and Algeria (Boudiar et al. 2020) displayed wider variability and consistently steeper and deeper root systems compared to commercial or elite cultivars. Several quantitative trait loci (QTLs) associated with root angle have been identified, including *qSRA-6A*, which influences root angle across diverse populations, indicating a conserved genomic region with potential for modern breeding (Kang et al. 2025). Interestingly, the *VERNALIZATION1* gene, known for regulating flowering time, also affects root angle in wheat and barley, with steep-root haplotypes prevalent in pre-Green Revolution and winter wheat varieties (Voss-Fels et al. 2018). This pattern suggests that steep-root ideotypes may have been historically favoured under marginal conditions, whereas shallow-root haplotypes were selected for high-input systems with abundant resources.

## Root angle trait is ecologically relevant

Root angle determines the spatial distribution of roots within the soil, enabling plants to access water and nutrients that are unevenly distributed across depth gradients. This trait is therefore not only a physiological characteristic but also an ecologically significant feature that shapes plant interactions with heterogenous soil environments and influences overall fitness and productivity in both natural and managed ecosystems. Australia provides an excellent natural system for investigating the ecological relevance of root angle due to its diverse climatic and edaphic gradients. Its three major cropping regions, southern low-rainfall, southern high-rainfall, and northern subtropical, represent global agroecological analogues, allowing insights gained from Australian systems to be broadly translated (Rao et al. 2023).

In the southern low-rainfall region (< 350 mm annual rainfall), sandy soils with high phosphorus in the topsoil and toxic element accumulation in subsoils favour shallow root angles that maximise early access to topsoil moisture and immobile nutrients. For example, the wheat genotype *RAC875* with a prominent convex hull area, showed improved phosphorus uptake under suboptimal phosphorus availability compared with other genotypes, such as Wyalkatchem (Nguyen and Stangoulis 2019). By contrast, the southern high-rainfall region (> 500 mm annual rainfall), characterised by compacted chromosols and vertosols, acidic topsoils, and frequent waterlogging, favours steep root angles that enable deep soil exploration. Consistently, wheat genotype *SeriM82*, with a steeper root angle, captured more deep soil moisture than the shallow-rooted Hartog under these conditions (Manschadi et al. 2010). The northern subtropical region, with variable rainfall, high temperatures, and deep vertosols, requires intermediate root angle phenotypes to balance topsoil phosphorus acquisition with deep soil moisture capture. Consistently, sorghum lines carrying *Stg1 QTLs*, which confer narrower root angles, have been shown to access up to 19 mm more water under drought stress (Borrell et al. 2014). However, deeper rooting in wheat and sorghum was shown to be advantageous only when topsoil phosphorus was sufficient, highlighting the need for intermediate phenotypes under low-phosphorus conditions (van der Bom et al. 2024; Hunter et al. 2024).

Collectively, these examples demonstrate how shallow root angles can confer advantages in low-rainfall, nutrient-stratified soils, steep angles enhance resource capture in high-rainfall environments, and intermediate phenotypes optimise performance under subtropical conditions. Capturing this variability under field conditions remains challenging yet essential for linking root traits to adaptive performance (Lombardi et al. 2025).

## Root angle is set in relation to gravity

**Fig. 2:**
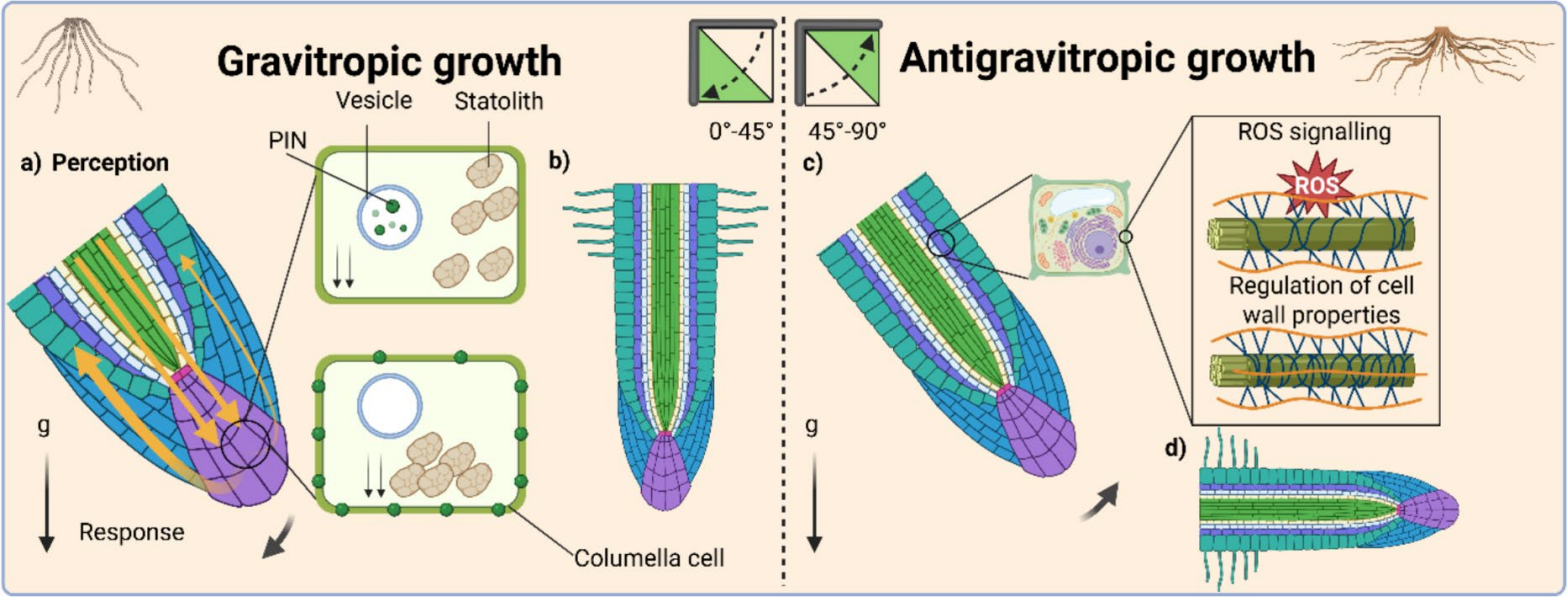
**Gravitropic and antigravitropic mechanisms driving the root angle set-point.** **a Perception:** Gravitropic growth is perceived by statoliths in the columella cells in the root tip (top columella cell). Dynamic interactions with vesicle membranes and the cytoskeleton trigger a signalling cascade altering the localization of auxin PINFORMED (PIN) efflux transporters (bottom columella cell). **Response:** PIN protein family trigger asymmetrical auxin polar transport in the root tip promoting root bending. **b.** Asymmetrical auxin polar transport promotes root gravitropism and affects root bending and downward growth. **c.** Antigravitropic growth through the anti-gravitropic offset (AGO) mechanism is affected by the up/down regulation of ROS signalling and cell wall loosening enzymes, (**d)** resulting in a non-vertical orientation. Image created with BioRender.com

Recent research has begun to uncover the fundamental molecular machinery that establishes root angle in cereals (reviewed in (Kirschner et al. 2024)). Setting the angle involves balancing two key molecular pathways, a gravitropic mechanism that guide roots downward in response to gravity, and the anti-gravitropic offset (AGO) mechanism, which counteracts this response to fine-tune across different classes and developmental stages (Fig. 2). Root angle is dynamic, shaped by the interplay of genetic and hormonal players that modulate growth over time and in response to biotic and abiotic environmental stresses encountered in soil.

Gravity perception initiates in the columella cells of the root cap, where amyloplast sedimentation acts as the primary stimulus. Amyloplasts, functioning as statoliths, are linked to the actin cytoskeleton, and their sedimentation alters the polarity of auxin-related PINFORMED (PIN) carriers, triggering differential auxin distribution (Band et al. 2012). Following perception, gravitropic signalling generates asymmetric auxin distribution, driving root bending toward the gravity vector. In rice, PIN2 re-localisation coincides with asymmetric expression of an auxin reporter after gravistimulation, suggesting this core auxin regulated mechanism is conserved in cereal crops (Yang et al. 2017; Xiong et al. 2025). Auxin redistribution is mediated by transporters such as OsAUX1 and OsPIN2, which direct auxin flow from the root cap to the elongation zone (Giri et al. 2018; Wang et al. 2018). Proteins such as VILLIN2 (OsVLN2) regulate actin filament dynamics, thereby influencing PIN recycling and auxin flow (Wu et al. 2015). In rice, DEEPER ROOTING 1 (DRO1) also regulates auxin-dependent asymmetric cell elongation, directly controlling root angle (Uga et al. 2013). Additional signalling components include the calcineurin B-like interacting protein kinase ZmCIPK15, which may act through calcium signalling, and ZmRSA3.2, which contributes to cytoskeletal reorganization (Ren et al. 2022; Schneider et al. 2022). Downstream, auxin-responsive genes such as *ZmRSA3.1* and *SOR1* mediate transcriptional and protein degradation pathways to enforce differential cell elongation and root bending (Chen et al. 2018; Ren et al. 2022).

In parallel, AGO mechanisms stabilise non-vertical growth angles, particularly in lateral, seminal, and crown roots (Fig. 2). While auxin–cytokinin crosstalk is implicated in AGO in *Arabidopsis* (Roychoudhry et al. 2023; Waidmann et al. 2019), cereal-specific components such as *ENHANCED GRAVITROPISM1* (*EGT1*) and *EGT2* represent auxin-independent AGO pathways (Kirschner et al. 2021; Fusi et al. 2022). These genes primarily function in the elongation zone by modulating reactive oxygen species (ROS) levels and cell wall mechanical properties. For example, *HvEGT1* encodes an F-box/Tubby domain protein that remodels cell wall structure via ROS signalling, reducing sensitivity to gravity-induced auxin asymmetry. Similarly, *EGT2* regulates ROS-related gene expression and cell wall dynamics in the elongation zone, pointing to a conserved AGO mechanism in cereals (Guo et al. 2023).

## Root angle regulation under environmental stresses

Although the core gravitropic-AGO regulatory framework is increasingly well understood, a major knowledge gap remains in how environmental stresses reprogram the balance between these pathways (Kirschner et al. 2024; van der Bom et al. 2025). Abiotic stresses modulate RSA, especially root angle through intricate hormonal crosstalk, particularly among auxin, ethylene, and abscisic acid as well as ROS dynamics and changes in cell wall remodelling (Fusi et al. 2022; Song et al. 2025; Xiong et al. 2025; Krieger et al. 2016; Joo et al. 2001; Cnodder et al. 2000). This interplay ensures that RSA remains responsive to water and nutrient status, as discussed below.

### Drought

**Fig. 3:**
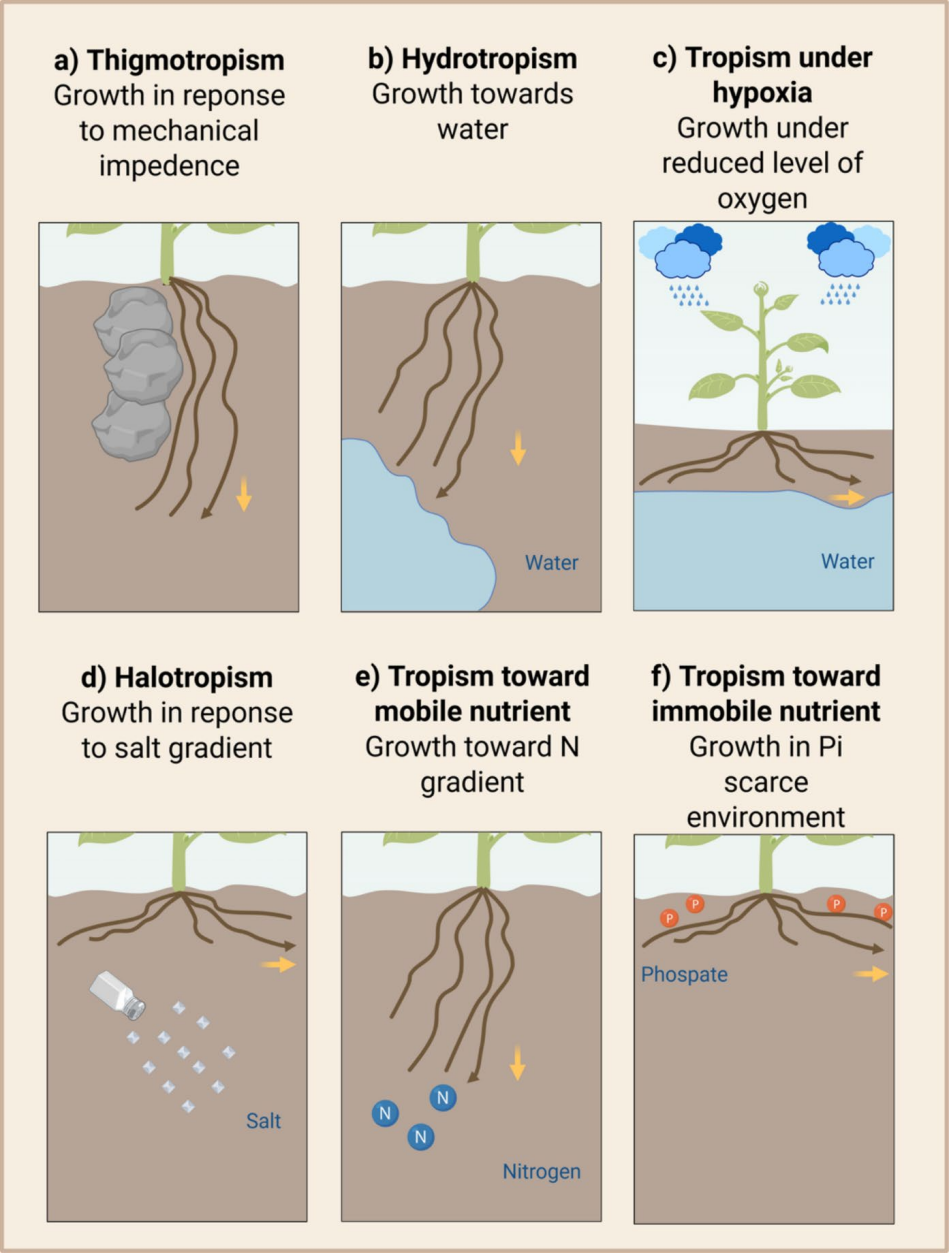
**Environmental factors and associated tropic responses that influence root angle.** Soil moisture not only determines water availability for roots, but also imposes mechanical, hydraulic, and chemical constraints on root growth. As soil dries, changes in its physical structure generate distinct stress domains that activate different tropic responses in roots, all of which interact with and may compete against the root angle set point. For example, **thigmotropism**, the bending response to touch or physical contact, can arise from obstacles or from highly compacted, very dry soil layers and is mediated through ethylene-ABA-auxin signalling (**a**) (Huang et al. 2022). **Hydrotropism**, the growth response to moisture gradients in soil, is shaped by ABA-auxin interactions and reflects root responses under moderately dry conditions where water is unevenly distributed across soil profiles and this response can compete with gravitropism (**b**) (Wexler et al. 2024). Conversely, excess soil moisture when water exceeds field capacity, leads to ethylene accumulation and **oxygen limitation**, both of which strongly influence root growth (**c**) (Waidmann et al. 2019). Superimposed on these water-related factors are additional tropic gradients: **salinity** (**d**) and in nutrient availability, mobile nutrients such as **nitrogen** (**e**) and relatively immobile nutrients such as **phosphate** (f), which elicit halotropic and other nutrient driven tropic responses (van der Bom et al. 2025). Image created using BioRender.com

Water availability is one of the most critical abiotic challenges limiting crop productivity, particularly in regions with seasonal water scarcity. RSA, and specifically root angle, plays a pivotal role in determining a plant’s capacity to access water stored in deeper soil layers (Fig. 3a, b). In water-limited environments, plants often exhibit a steep root angle phenotype, which facilitates deeper soil penetration and access to residual moisture (Schenk and Jackson 2005). This trait is especially advantageous in warm-temperate and tropical ecosystems where water tends to accumulate at depth due to gravitational percolation and surface evaporation. Conversely, in arid regions with sporadic and shallow rainfall, a shallow root angle may be more adaptive, allowing plants to exploit transient surface moisture (Ogura et al. 2019). Thus, optimal root angle is highly context-dependent, shaped by the spatial and temporal distribution of water in the soil.

A key genetic determinant of drought-adaptive root angle is *DRO1* in rice, which enhances asymmetric cell elongation in the root tip, promoting a steeper angle. Overexpression of *DRO1* in shallow-rooting cultivars significantly improves drought tolerance and yield under water-limited conditions (Uga et al. 2013). Similarly, in maize, ZmCIPK15 modulates root angle in response to nitrogen and drought stress, highlighting the integration of nutrient and water signaling in shaping RSA (Schneider et al. 2022).

Recent findings by Xiong et al. (2025) elucidate a hormonal cascade linking drought perception to root architectural adaptation in cereals. Under drought, ABA accumulates in the root tip and acts upstream of auxin biosynthesis, enhancing the gravitropic response and promoting a steeper root angle (Xiong et al. 2025). ABA-deficient rice mutants fail to adopt a steeper angle under drought, maintaining a shallow RSA similar to well-watered conditions. These mutants exhibit reduced auxin levels and impaired gravitropic bending, which can be rescued by exogenous auxin application, confirming that ABA acts upstream of auxin in this regulatory pathway. Auxin reporter assays further demonstrate that drought-induced ABA accumulation promotes asymmetric auxin distribution in the root epidermis, a hallmark of gravitropic bending response machinery. In field trials with soybean, hydrotropism and gravitropism interact and compete depending on the intensity of the stimuli (Tsutsumi et al. 2002). Eapen et al. 2017 showed that a robust hydrotropic response displayed reduced gravitropic curvature when compared to a weak hydrotropic response (Eapen et al. 2017). This result may indicate that in some particular cases a strong hydrotropic response could be achieved only if the gravity response weakens. In *Arabidopsis*, hydrotropism i.e., growth toward water gradient is mediated by ABA signalling, which suppresses gravitropic auxin redistribution and promotes cortical elongation via SnRK2 kinases and the MIZ/GNOM pathway (Dietrich et al. 2017; Miyazawa et al. 2009).

Together, these findings highlight a dynamic ABA–auxin signaling network that fine-tunes root angle plasticity in response to soil water availability, offering promising molecular targets for breeding drought-resilient cereal crops.

### Flooding and waterlogging

Flooded or waterlogged soils create hypoxic conditions that severely limit oxygen availability to roots, impairing respiration and growth (Sasidharan et al. 2018) (Fig. 3c). In response, plants rapidly synthesise ethylene, a key hormonal signal that mediates adaptive responses to low oxygen. Ethylene accumulation under hypoxia inhibits elongation and promotes upward root bending, resulting in a shallower root angle (He et al. 2024; Waidmann et al. 2019). This shallow rooting response is thought to help roots escape oxygen-deprived zones and access more aerated soil layers. Ethylene also promotes aerenchyma formation and the development of adventitious roots, which enhance internal oxygen diffusion and support root survival in anoxic conditions (Voesenek and Bailey-serres 2015). Notably, rice cultivars carrying the *qSOR1* allele exhibit improved waterlogging tolerance by maintaining shallow rooting and avoiding hypoxic zones (Kitomi et al. 2020).

Recent work in rice and maize has revealed that ethylene regulates root angle through crosstalk with auxin signalling. Kong et al. (2024) demonstrated that ethylene-insensitive mutants exhibit impaired gravitropic responses and shallower root systems under control conditions, highlighting the role of ethylene in modulating auxin biosynthesis and distribution (Kong et al. 2024). This suggests that ethylene perception is essential for directing auxin-mediated root angle regulation under control conditions. However, how this interaction is modulated under flooding or waterlogging remains unknown. Future studies are needed to determine whether these mutants can adapt their root angle appropriately under hypoxic conditions or fail to do so.

### Salt Stress

Salinity stress disrupts cellular water balance and ion homeostasis, leading to reduced root elongation and altered RSA (Fig. 3d). Under salt stress, roots often exhibit a shallower growth angle, a response known as halotropism, which allows them to avoid saline zones in the soil (Kitomi et al. 2020). This response is mediated by changes in auxin transport and signaling, as well as the accumulation of ROS that influence cell wall properties (Zhao et al. 2022). In rice, the transcription factor OsMADS25 regulates salt tolerance by modulating the expression of *R3L1*, a gene involved in ROS homeostasis and cell wall remodelling. Loss-of-function mutants in *R3L1* show impaired lateral root formation and reduced salt avoidance, while overexpression enhances shallow rooting and salt tolerance (Zhao et al. 2022).

Further supporting this regulatory network, ABA signaling has been shown to gate salt-induced root responses. Lamers et al. (2025) demonstrated that ABA acts as a regulatory switch that suppresses sodium-specific transcriptional responses in *Arabidopsis* roots (Lamers et al. 2025). ABA-deficient or signaling-impaired mutants show prolonged salt-induced gene expression, increased root swelling and cellular damage and failure to redirect root growth away from high sodium concentrations, underscoring the importance of ABA in modulating root angle plasticity under salt stress.

Together, these findings reveal that salt stress reshape root angle through a complex network involving ABA signaling, ROS dynamics, cell wall remodelling, and auxin-dependent and independent pathways, enabling plants to adaptively reorient their roots and mitigate stress exposure.

### Nutrient availability related stresses

Nutrient deficiencies in soils, such as low availability of nitrogen and phosphate, lead to reduced plant growth and thereby ultimately to yield losses (Sun et al. 2024) (Fig. 3e and f). Since mineral nutrients in the soil are not evenly distributed, the placement of the roots in different layers is crucial to provide access to the nutrients in different soil layers (Franzluebbers and Hons 1996). Often, phosphorus (P), potassium (K), and micronutrients are located at the topsoil, while nitrogen can be found at the topsoil or in deeper soil layers due to leaching (Jobbágy et al. 2001). In response to nutrient availability, plants are able to alter their root system architecture, including the root angle (recently reviewed in (van der Bom et al. 2025). For example, low phosphate conditions induce a shallower crown root angle in rice, via the actin-binding protein Rice Morphology Determinant (RMD) (Huang et al. 2018). (Li et al. 2025) showed that rice plants carrying a mutation in the *GRAVITROPISM LOSS 1* gene exhibit a shallower root phenotype and take up more nutrients from the topsoil compared to plants with steeper root systems. This suggests that a shallower root architecture can be advantageous when nutrient availability is concentrated in the upper soil layers.

Under low nitrogen conditions, the root angle of maize brace and crown roots becomes steeper (Trachsel et al. 2013; Schneider et al. 2022). A steeper and deeper root system can improve nitrogen uptake by reaching into deeper soil layers with available nitrogen (Schneider et al. 2022). This plasticity in root angle in response to nutrient availability implies that the root system adapts for a better access to the scarce nutrients.

## Emerging implications of the cell wall as a converging point in root angle regulation

Plant cell walls provide mechanical strength and serve as primary site for perceiving and responding to environmental signals (Hoson and Wakabayashi 2015). While the role of auxin and its distribution in regulating differential growth during gravitropic responses is well established, the mechanistic links between cell wall properties and root bending remain less understood (Jonsson et al. 2023).

Evidence suggests that root bending is regulated through the interaction between auxin-driven signaling and cell wall mechanics. The primary cell wall consists of cellulose fibrils cross-linked with hemicellulose embedded in a pectin matrix. In *Arabidopsis*, differential auxin distribution between the upper and lower side of a root, regulated by gravity, induces Ca^+2^-dependent acidification of the cell wall on the upper side (Monshausen et al. 2011; Barbez et al. 2017). This acidification activates cell wall remodelling proteins such as expansins, which loosen the cell wall by cleaving cross-links of cellulose-hemicellulose (Zhang and Hasenstein 2000). Conversely, alkalinization on the lower side promotes cell wall stiffening, leading to reduced growth (Barbez et al. 2017). These coordinated responses create differential cell elongation and initial curvature, thereby determining the final growth angle of the root (Jonsson et al. 2023).

ROS further integrate into this process as key signalling components shaping RSA (Bellucci et al. 2024). In *Arabidopsis,* gravity-dependent auxin redistribution induces ROS production, which accumulates in the lower bending region, inhibiting elongation by promoting cell wall stiffening (Krieger et al. 2016; Joo et al. 2001). Similarly, in both maize and soybean, changes in gravity direction lead to auxin accumulation on the lower side of the root, resulting in localised ROS accumulation in the same region (Tang et al. 2024). De Cnodder et al. further demonstrated that ROS promote cross-linking of structural cell wall proteins during ethylene precursor-induced inhibition of elongation in *Arabidopsis* root epidermal cells (Cnodder et al. 2000), reinforcing the link between ROS and cell wall integrity during gravitropic responses.

Recent studies in cereals provide emerging support for similar processes. In barley, the *EGT1* gene, functioning in AGO machinery, alters ROS homeostasis and cell wall stiffening processes to control root angle (Fusi et al. 2022). In rice, activation of *Increased Leaf Angle1* (*OsILA1*) expression by auxin response factors (ARFs) OsARF12 and OsARF25 in the lower side of the epidermis of roots during the gravitropic response enhances cell wall thickness, inhibiting cell elongation and promoting bending (Song et al. 2025). These findings indicate that cereals may employ analogous auxin-ROS‑mediated modifications of cell wall mechanics, although the underlying biochemical pathways remain less fully characterised than in *Arabidopsis*.

Collectively, these findings indicate that the balance between gravitropic and anti-gravitropic mechanisms translate upstream environmental signals into differential cell elongation, thereby shaping the root angle. Gravitropic mechanisms rely on auxin-dependent signal transduction, cytoskeletal organization and ROS dynamics, whereas antigravitropic mechanisms involve ROS mediated cell wall stiffening. Taken together, these developmental pathways, combined with environmental responses that modulate ROS production and wall mechanics, ultimately converge at the cell wall. Thus, the cell wall emerges as a critical downstream integrator of developmental and environmental stress pathways, providing a mechanistic framework for understanding molecular regulation of root angle and thus RSA plasticity.

However, it is important to emphasise that the processes described in this framework are based partly on findings from *Arabidopsis* and only partly on emerging evidence from cereals. As a result, the cell wall should currently be viewed a promising but still partially validated convergence point for integrating developmental and environmental cues in cereal roots, and its mechanistic role requires further targeted investigation across species, root types, and stress contexts.

## Future directions and open questions

Despite significant progress in understanding root angle regulation in recent years, several critical gaps remain, especially concerning its regulation under environmental stresses. Most current studies focus on a single stress, whereas real world conditions involve complex combinations of drought, salinity, nutrient limitation, and temperature extremes. How these combined stresses interact with the underlying gravitropic and antigravitropic machinery to influence root angle regulation is still poorly understood.

A particularly compelling unresolved problem is how root systems integrate opposing stress signals that demand conflicting root angle responses. For example, drought promotes deeper rooting with steeper root angles in cereals to access subsoil water, mediated in rice by an ABA dependent local auxin biosynthesis cascade that enhances gravitropic response (Xiong et al. 2025). In contrast, phosphate deficiency in rice promotes a shallower root angle for topsoil foraging, partly by increasing levels of the actin binding protein RMD, which strengthens the interaction between actin filaments and statoliths. This enhanced interaction slows statolith sedimentation, thereby weakening auxin driven gravitropic response and resulting in shallower crown roots (Huang et al. 2018). Intriguingly, both responses converge on the same auxin redistribution component to direct root angle in opposite directions. Whether the drought induced ABA-auxin pathway or the phosphate-starvation driven RMD-actin-auxin pathway is more strongly or differently modulated under combined stress, and whether this modulation depends on stress severity, timing, order of occurrence, root class or tissue specificity remains entirely unknown in cereals. Functional evidence indicates that deeper rooting in durum wheat under drought confers a yield benefit only when topsoil phosphorus is sufficient and this advantage disappears under simultaneous phosphate limitation (van der Bom et al. 2024).

Similar converging mechanisms have been observed in *Arabidopsis*, analysing the antagonism between gravitropism and hydrotropism. In *Arabidopsis*, ABA-dependent signalling through the MIZ1/MIZ2 pathway in cortical cells actively attenuates gravitropic auxin redistribution when roots encounter a water potential gradient, establishing a context-dependent hierarchy between the two tropic responses (Dietrich et al. 2017; Wexler et al. 2024). Whether an analogous hierarchical mediation mechanism operates in cereal roots, which differ markedly from the *Arabidopsis* in root system organisation in terms of auxin transport components and AGO machinery, remains to be demonstrated. Evidence on variability in root angle and tropic responses in cereals remains limited, particularly across different root types such as seminal and crown roots. Are these responses consistent across root classes?

Addressing these questions is also constrained by current phenotyping approaches, which largely focus on seedlings under controlled conditions and fail to capture temporal and spatial variability in the field. Importantly, resolving these gaps will require studying root angle regulation directly in real soil environments, where mechanical resistance, moisture gradients, and heterogeneity naturally shape tropic responses in ways that challenges replication in artificial growth systems. Advances in scalable, high-throughput phenotyping and imaging technologies are urgently needed to monitor root angle dynamics across root types under realistic field conditions. Studies on crown roots, in particular, remain resource-intensive, highlighting the need for innovative and cost-effective strategies to link root traits with adaptive performance. However, extracting root trait data remains challenging because segmenting roots from the surrounding soil matrix remains a notoriously time-consuming limiting step, restricting the throughput required for phenomics studies, especially for adventitious and crown root traits. Overcoming this bottleneck will require substantial advances in AI-based segmentation pipelines for cereal roots to achieve automated, reliable, and potentially field-scalable root segmentation.

## Conclusion

Root angle is emerging as a critical trait for improving crop resilience and productivity under increasingly variable environmental conditions. Its ecological relevance, genetic variability, and responsiveness to environmental cues underscore its potential as a breeding target. Recent advances have revealed that root angle is regulated by a complex interplay of gravitropic and antigravitropic mechanisms, hormonal signaling networks, and stress-responsive pathways. Importantly, these upstream signals seem to converge at the cell wall, which acts as a critical integrator translating environmental signals into molecular regulation of root angle and RSA plasticity.

Despite these insights, major knowledge gaps remain. How multiple tropic responses coordinate under combined stresses, and how these responses vary across root classes, are unresolved questions. Furthermore, current phenotyping approaches fail to capture dynamic root angle adjustments under field conditions, limiting our ability to link trait plasticity with adaptive performance. Addressing these challenges will require innovative, high-throughput phenotyping platforms, integrative molecular studies, and systems-level approaches to exploit root angle for climate-resilient agriculture. Overall, harnessing root angle as a breeding target offers a promising avenue to optimise resource acquisition, reduce input dependency, and enhance crop productivity in the face of global climate change.
